# The role of family support in the self-rated health of older adults in eastern Nepal: findings from a cross-sectional study

**DOI:** 10.1186/s12877-023-04619-1

**Published:** 2024-01-04

**Authors:** Aman Shrestha, Saruna Ghimire, Jennifer Kinney, Ranju Mehta, Sabuj Kanti Mistry, Shoko Saito, Binod Rayamajhee, Deepak Sharma, Suresh Mehta, Uday Narayan Yadav

**Affiliations:** 1 Department of Sociology & Gerontology and Scripps Gerontology Center, Miami University, Oxford, OH USA; 2 Little Buddha College of Health Sciences, Kathmandu, Bagmati Nepal; 3 Centre for Primary Health Care and Equity, University of New South Wales, Sydney, Australia; 4 School of Optometry and Vision Science, Faculty of Medicine and Health, UNSW, Sydney, Australia; 5 School of Health Sciences, University of Tasmania, Hobart, Tasmania Australia; 6 Koshi Province Ministry of Health, Biratnagar, Koshi Nepal; 7 National Centre for Epidemiology and Population Health, The Australian National University, Canberra, ACT Australia

**Keywords:** Filial piety, Self-reported health, Subjective health, Family-based care, Informal care, Social support

## Abstract

**Background:**

Nepal’s low fertility rate and increasing life expectancy have resulted in a burgeoning older population. For millennia, filial piety shaped family cohesion and helped Nepali older adults achieve positive outcomes, but recently, it has been eroding. Furthermore, there are not enough institutional support options or alternatives to family-based care to deal with the biosocial needs of older adults. This study explored the association between family support and self-rated health among Nepali older adults.

**Methods:**

A community-based cross-sectional survey in eastern Nepal’s two districts, Sunsari and Morang, interviewed 847 older adults (≥ 60 years). The final analytical sample was 844. Participants were asked whether they received assistance with various aspects of daily life and activities of daily living from their families. Multivariable logistic regression examined the association between family support and self-rated health.

**Results:**

Participants who received support with various aspects of daily life had 43% higher odds of good health, but after adjusting for control variables, the result only approached statistical significance (*p* = 0.087). Those who received family assistance with activities of daily living had nearly four times higher odds (*OR*: 3.93; 95% *CI*: 2.58 – 5.98) of reporting good health than participants who lacked this support.

**Conclusions:**

Given the important role of family support in Nepali older adults’ health, government programs and policies should create a conducive environment to foster family-based care until more comprehensive policies for older adults’ care can be put into effect. The results of this study can also help shape the global aging environment by highlighting the need for family support in older care, particularly in low-income nations with declining traditional care systems and weak social security policies.

**Supplementary Information:**

The online version contains supplementary material available at 10.1186/s12877-023-04619-1.

## Background

Nepal’s demographic transition with declining fertility and mortality rates mirrors the global trend, resulting in a noticeable growth in its older population. For example, the older population increased from 5.88% in the 1971 census to 10.21% in the 2021 census [1, 2]. Furthermore, the older population’s growth rate surpassed the nation’s average population growth [1, 2]. With the aging of the population comes the need to address its expanding social and healthcare needs. However, Nepal has been unable to meet such needs due to financial and policy constraints [3].

Aging at the individual level results in physical and physiological changes that impact health and well-being. Self-rated health is a widely used tool in social science research that captures individuals’ perceptions and experiences of their health. It is one of the most widely used health and well-being indicators in general population surveys [4–7]. It also strongly predicts many health outcomes, including morbidity, mortality, functional limitations, and cognitive disorders [8–10]. As a reliable measure to capture the population’s general health, self-reported health has been employed in various national surveys across the globe and used in various languages and cultures.

Previous researchers have used self-rated health as an important indicator in understanding the subjective health and well-being of older adults [4, 11–13]. Some studies conducted in Nepal found that older adults’ self-rated health was associated with increased physical activity [14], higher life satisfaction [15], an absence of chronic health problems [16], and shorter or no migration history [17]. However, no prior study has specifically studied the self-rated health and well-being of Nepali older adults in the context of family support. Earlier studies not limited to older adults explored family support in the context of health care utilization, mental health, and adherence to diet [18–20]. Studies conducted in India and China, bordering countries that share similar cultural contexts to Nepal, also found a positive association between family support and older adults’ subjective health [11, 21, 22]. Further research conducted in other nearby countries in South Asia such as Pakistan [23, 24], Bhutan [25] and Sri Lanka [26, 27], that have a comparable culture but distinct religious differences with Nepal also showed improved health and well-being when family support was present. A 2019 systematic review based on studies from eight Asian countries – China, Japan, Singapore, Taiwan, Hong Kong, Korea, Macau and Thailand also concluded that family support is associated with reduced depression among community dwelling older adults [28].

Nepali families are characterized by a collectivist orientation, multigenerational living arrangements, and high solidarity. Family-based caregiving in Nepal resembles practices similar to those of South Asian countries [29], whereby adult children – specifically the son and daughter-in-law – ultimately become socially, legally, and morally responsible for caring for older parents’ holistic needs [30]. Such caregiving in Nepal is influenced by Hindu filial piety, selfless service, and religious-moral duties [31]. Indeed, filial piety, a practice of caring for and supporting parents, has been practiced for centuries, and intergenerational support has been the backbone of the old-age care system in Nepal [32]. However, the rise in nuclear families and high out-migration of adult children has shifted the family structure from multigenerational to nuclear [33], challenging family-based care and support for older members in Nepal.

Nepal’s government is in the early stages of developing programs and policies to support the diverse needs of its burgeoning older population. However, developing such policies requires evidence, which is currently lacking for most care issues that target older adults. Given that the principle of filial piety is deeply rooted in Nepali society, where it is both a tradition and a legal requirement, exploring the role of family support in older adult’s health in light of changes in family structure is an important currently unstudied research domain.

This study conceptualizes the support older family members receive from their adult children from a collectivist approach where the members living in a group complement each other’s needs. Bengtson and colleagues’ intergenerational solidarity model identifies six dimensions of collectivistic families: association (high frequency and patterns of interaction), affection (positive sentiments), consensus (agreement on attitudes, values, and beliefs), functional (helping and exchange of resources), normative (commitment to fulfilling family roles and obligations) and structural (number, type and proximity of family members) [34, 35]. Although originally developed in the context of US and Western European families, the model has been used to examine family relationships in other cultures [36, 37]. Bengtson and colleagues’ model is useful in “unpacking” the types of support that are provided to older family members in the Nepali context. Consistent with filial piety and guided by Hindu beliefs, adult children assist older parents as a sign of respect and social obligation, as described in the solidarity model. Likewise, co-residence in multigenerational households fosters greater affection and daily interactions and facilitates the exchange of resources between older adults and their adult children. Older parents are revered in Nepali culture, and family members usually exhibit strong bonds and closeness to one another while sharing common customs and beliefs.

This research addresses two important gaps in literature. First, family support in earlier studies conducted in Nepal was not conceptualized through the lens of filial piety and caregiving. Second, most prior studies from Nepal have been conducted in urban settings, even though approximately 83% of the total population of Nepal lives in rural areas [1].

This study explores how being reliant on family members for aspects of daily life and activities of daily living is related to self-rated health in rural settings of eastern Nepal. The study hypothesized that older adults who received more of the two types of support from their families would report better (self-reported) health. The findings from this research conducted in rural setting will help clarify how urbanization and migration are gradually upending the family-based older care system in low-income countries or countries with family based informal care system, and its implications in global aging issues. In the Western context, where there has been a growing preference for de-institutionalization [38] and aging in place [39] including integrated care approaches [40], paid informal care [41], and paid leave for family caregivers of older adults [42], this study will also help in explaining the essence of family support towards older family members. Further, this study will add valuable contribution to the literature that can guide the models of care in countries with comparable but changing family traditions and living arrangements; inadequate social security; and developing policies and aging services for older adults.

## Methods

### Study design and study population

A community-based cross-sectional study conducted between July and September 2020 surveyed 847 older adults 60 years and older from rural settings in two districts in eastern Nepal, Sunsari and Morang (Supplementary Fig. A). The demographic and socioeconomic characteristics of the study districts and the national average are provided in Supplementary Table 1. To summarize, the five development regions of Nepal are Eastern (EDR), Central, Western, Mid-Western, and Far Western. The aging index of the Eastern Development Region (16.2) is ranked the second highest after the Western (18.75) [1]. Ethnic composition in EDR is very diverse comprising major castes and ethnic groups [1, 43]. An earlier study showed that EDR, particularly the Tarai regions including the study districts, ranked high in most of the development indicators including economic, social, infrastructure and governance [44]. Thus, EDR has been an important contributor in terms of both social as well as economic growth of Nepal.

Using multi-stage sampling, two rural municipalities from each district were randomly selected, followed by a random selection of four wards in each rural municipality. Sampling frames were obtained from ward offices of related rural municipalities, and samples were randomly chosen proportionate to population size. Nepali adults aged 60 years or above, who were willing to participate, understood the instructions, and provided informed consent were eligible to participate in this survey. Those living in care institutions, who were cognitively impaired, had a hearing impairment, or could not communicate were excluded from this survey. No proxies were employed for interviewing.

### Sample size

A sample size of 770 was calculated using the formula $\left[\frac{{{\varvec{Z}}}^{2}}{4{{\varvec{e}}}^{2}}\right]$ where: Z represents the z-score corresponding to the chosen confidence level (i.e., 1.96 for 95% confidence) and e represents the margin of error (i.e., 0.05 for a ±5% margin of error); the estimated prevalence was unknown and thus, was conservatively set at 50%. An additional 10 percent non-response rate finally yielded 847 number of samples for this study.

### Data collection

Trained enumerators conducted face-to-face interviews using the KoBo toolbox survey software application (pre-installed on tablets) [45]. Each interview took approximately 35 min to complete, and all questions related to variables used in this study were closed-ended. The tool for this study was prepared in English but converted to the Nepali language to facilitate the data collection process. Due to the onset of COVID-19 at the time of this survey, questionnaires were pilot tested on volunteers similar to the anticipated cohort concerning demographic characteristics, and minor editorial revisions were made to the questionnaire.

### Variables

#### Dependent variable

Self-rated health was assessed by asking, “In general, how do you rate your health?”. The response choices were “very good”, “good”, “medium”, “poor”, and “very poor”. For this study, the 5-response Likert scale was dichotomized by merging “very good” and “good” as “good” and the remaining three as “poor”, consistent with earlier research [9, 46]. Prior studies documented the validity of this single item to assess subjective health [47, 48]. Specifically, it has good face validity [49], criterion validity [50], and predictive validity with mortality in several studies conducted in India [4, 51].

#### Independent variables

Family support was operationalized as adult children providing financial, instrumental, and emotional support for older parents. Two specific types of family support were examined: 1) family support with various aspects of daily life and 2) family assistance with daily activities. Family support with various aspects of daily life was assessed by the question, “Do you depend on family for various aspects of daily life?” and the family assistance with activities of daily living was based on the question, “Do you depend on family for activities of daily living?” The response choice for each question were “yes” or “no”. The first concept included support for the necessities of life, including food, housing, transportation, and medical expenses, whereas the latter referred to assistance with activities of daily living (ADLs) and instrumental activities of daily living (IADLs).

#### Control variables

Control variables were age (in years), sex (male or female), marital status, education status, religion (Hindu vs. non-Hindu), ethnicity, occupation, family structure, district of residence, and morbidity status. Marital status was dichotomized as “married” for those who were married and “without a partner” for the remainder of the participants (i.e., unmarried, divorced, separated, and widowed). Those who could not read or write in Nepali were categorized as having “no education”; “non-formal education” included individuals who had attended classes or training provided by an education or training institution and thus could read and write in Nepali but did not attend formal school [52]. The last category, the “formal education” group, included those who attended schools. Similar approach has been used in previous studies from Nepal [53, 54].

Ethnic groups were identified using Nepal’s Integrated Health Management Information System classification [55] and were recategorized into three groups, specifically “Dalit and religious minority”, “disadvantaged caste”, and “upper or advantaged caste”. The first two groups are considered relatively disadvantaged in Nepal. The occupation was recoded into four categories: “unemployed”, “agriculture”, “business or employed”, and “retired and others”. Family structure was categorized as “nuclear family” or “multigenerational family”. The district of residence (Morang or Sunsari) was also included as a control. The existence of seven non-communicable diseases or health problems (high blood pressure, osteoarthritis, heart disease, diabetes, chronic obstructive pulmonary disease, stroke, and fractured hand/leg) was assessed, and a cumulative score was calculated that ranged from 0 to 7, with 0 indicating no disease or health condition and 7 indicating the presence of all seven diseases or health conditions. Finally, the cumulative score was categorized to create a morbidity variable with three categories – no morbidity, single morbidity, and multimorbidity.

### Data management and statistical analysis

Data were cleaned and analyzed using SAS Version 9.4 [56]. Under multicollinearity checks, all variables had a variance influencing factor (VIF) of less than 2.5. Thus, multicollinearity was not a concern, and all independent variables in Table 1 were included in the final model. The logistic regression tested for the deletion diagnostics, which determined whether removing a specific observation causes a significant change to the estimates [57]. As a result, two influential observations were removed. Further, family support for various aspects of daily life had one missing data case; therefore, that observation was excluded from the study. Hence, the final analysis included 844 participants.

**Table 1:** Demographic and socioeconomic characteristics of older adults by self-reported health status

Characteristics	Total sample *n* = 844	Self-reported health		*P*-value
		Good	Poor	
		*n* = 597 (70.7%)	*n* = 247 (29.3%)	
	n (%)	n (%)	n (%)	
*Primary predictors*				
**Family support with various aspects of daily life**				0.024^*^
No	193 (22.9)	124 (20.8)	69 (27.9)	
Yes	651 (77.1)	473 (79.2)	178 (72.1)	
**Family assistance with activities of daily living**				< 0.001^***^
No	456 (54.0)	286 (47.9)	170 (68.8)	
Yes	388 (46.0)	311 (52.1)	77 (31.2)	
*Control variables*				
**Age (mean ± SD)**	68.0 ± 7.2	66.8 ± 6.8	70.8 ± 7.2	< 0.001^***^
**Sex**				0.443
Male	468 (55.5)	326 (54.6)	142 (57.5)	
Female	376 (44.5)	271 (45.4)	105 (42.5)	
**Marital status**				< 0.001^***^
Married	644 (76.3)	485 (81.2)	159 (64.4)	
^a^Without partner	200 (23.7)	112 (18.8)	88 (35.6)	
**Education**				0.066
No education	469 (55.6)	347 (58.1)	122 (49.6)	
Non-formal education	284 (33.6)	190 (31.8)	93 (37.8)	
Formal education	91 (10.8)	60 (10.1)	31 (12.6)	
**Religion**				0.003^**^
Hindu	806 (95.5)	562 (94.1)	244 (98.8)	
Non-Hindu	38 (4.5)	35 (5.9)	3 (1.2)	
**Ethnicity**				0.004^**^
Upper or advantaged caste groups	129 (15.3)	82 (13.8)	47 (19.0)	
Dalit and religious minority caste groups	162 (19.2)	103 (17.2)	59 (23.9)	
Disadvantaged caste groups	553 (65.5)	412 (69.0)	141 (57.1)	
**Occupation**				0.025^*^
Unemployed	354 (41.9)	252 (42.2)	102 (41.3)	
Agriculture	166 (19.7)	122 (20.5)	44 (17.8)	
Business or employed	95 (11.3)	76 (12.7)	19 (7.7)	
Retired and others	229 (27.1)	147 (24.6)	82 (33.2)	
**Family structure**				0.321
Nuclear	155 (18.2)	114 (19.1)	40 (16.2)	
Multigenerational	690 (81.8)	483 (80.9)	207 (83.8)	
**District of residence**				0.087
Sunsari	435 (51.5)	278 (46.6)	131 (53.0)	
Morang	408 (48.5)	319 (53.4)	116 (47.0)	
**Morbidity**				< 0.001^***^
No morbidity	437 (51.8)	330 (55.3)	107 (43.3)	
Single morbidity	261 (30.9)	180 (30.1)	81 (32.8)	
Multiple morbidities	146 (17.3)	87 (14.6)	59 (23.9)	

*SD* Standard deviation

^*^p < 0.05, ^**^p < 0.01, ^***^p < 0.001

^a^Without partner category includes unmarried, divorced, separated, and widowed

Univariate statistics were generated for each variable. Multivariable logistic regression models analyzed the relationship between the explanatory and outcome variables. Adjusted and unadjusted odds ratios (OR) and 95% confidence intervals (95%CI) are reported in Table 2. The concordance statistics (c-statistics) determined the predictability of the model. The range of c-statistics includes 0.5 to 1, with a higher value denoting higher predictive power [58].

**Table 2:** Factors associated with odds of better self-reported health

Characteristics	Unadjusted	Adjusted^a^
	OR (95%CI)	OR (95% CI)
*Primary predictors*		
**Family support with various aspects of daily life** (ref = no)		
Yes	1.43 (1.05–2.08)^*^	1.56 (0.94–2.61)
**Family assistance with activities of daily living** (ref = no)		
Yes	2.40 (1.76–3.29)^***^	3.93 (2.58–5.98)^***^
*Control variables*		
**Age**	0.93 (0.91–0.95)^***^	0.91 (0.89–0.94)^***^
**Sex** (ref = male)		
Female	1.12 (0.83–1.52)	0.94 (0.61–1.45)
**Marital status** (ref = without partner^b^)		
Married	2.40 (1.72–3.34)^***^	2.04 (1.35–3.10)^***^
**Education** (ref = no education)		
Non-formal education	0.71 (0.52–0.99)^*^	0.49 (0.32–0.76)^**^
Formal education	0.68 (0.42–1.10)	0.46 (0.24–0.86)^*^
**Religion** (ref = non-Hindu)		
Hindu	0.20 (0.06–0.65)^**^	0.10 (0.03–0.41)^**^
**Ethnicity** (ref = upper caste groups)		
Dalit and religious minority caste groups	1.00 (0.62–1.62)	0.54 (0.28–1.03)
Disadvantaged caste groups	1.68 (1.12–2.52)^*^	1.37 (0.81–2.32)
**Occupation** (ref = unemployed)		
Agriculture	1.12 (0.74–1.70)	1.03 (0.56–1.89)
Business or employed	1.62 (0.93–2.81)	1.32 (0.63–2.77)
Retired and others	0.73 (0.51–1.04)	0.53 (0.32–0.86)^*^
**Family structure** (ref = nuclear)		
Multigenerational	0.82 (0.55–1.22)	0.56 (0.34–0.89)^*^
**District of residence** (ref = Sunsari)		
Morang	1.30 (0.96–1.74)	1.57 (1.02–2.43)^*^
**Morbidity** (ref = no morbidity)		
Single morbidity	0.48 (0.32–0.71)^***^	0.52 (0.35–0.78)^**^
Multiple morbidities	0.72 (0.51–1.01)	0.40 (0.24–0.65)^***^

*Ref* Reference category, *OR* Odds Ratio, *CI* Confidence Interval

^*^p < 0.05, ^**^p < 0.01, ^***^p < 0.001

^a^Adjusted for all the variables in the table. Fit statistics for final adjusted model: generalized *R*-square = 0.21; Max-rescaled *R*-square = 0.29; c-statistic = 0.79

^b^Without partner category includes unmarried, divorced, separated, and widowed

## Results

### Participant’s characteristics

Table 1 shows the descriptive findings, overall, and by participant’s self-reported health status. The majority of the participants reported having good health (70.7%). Approximately three-fourths (77.1%) received support from their family with various aspects of daily life, and almost half (46.0%) with activities of daily living. Among participants reporting good health, approximately 79% received family support with various aspects of daily life, and 52% received assistance for activities of daily living (Table 1).

The mean age of participants was 68 years (*sd* = *7.2*). The majority of the participants were male (55.5%), married (76.3%), with no education (55.6%), and Hindu (95.5%). Participants were predominantly from disadvantaged caste groups (65.5%), unemployed (41.9%), and lived in multigenerational families (81.8%). Study participants were fairly distributed by study districts; 51.5% were from Sunsari, and 48.5% were from Morang. Almost half of the participants had either a single morbidity (30.9%) or multiple morbidities (17.3%) (Table 1).

### Association between family support and self-rated health

Table 2 provides odds ratios and significance levels for both unadjusted and adjusted models. The c-statistics (0.79) for the final model (Table 2) suggested marginally good predictive power for the model. Both types of family support were significantly associated with higher odds of self-reported health in the unadjusted model. Participants who received support with various aspects of daily life had 43% and 56% higher odds of reporting good health in the unadjusted and adjusted models, respectively. However, the odds ratio was no longer statistically significant when adjusted for covariates, although the adjusted results approached statistical significance (*p* = 0.087). Likewise, after adjusting for covariates, those who received family assistance with activities of daily living were almost four times more likely to report good self-rated health (*OR* = 3.93; 95% *CI*: 2.58–5.98) (Table 2).

### Covariates and self-rated health

Each additional year of age resulted in a 9% decrease in odds of good health (*OR* = 0.91; 95% *CI:*0.89–0.94), holding all other covariates constant. After controlling for covariates, the odds of reporting good health were two times greater for married participants than those without a partner (*OR* = 2.04; 95%*CI*: 1.35–3.10). Compared to those without education, those with formal (*OR* = 0.46) and non-formal (*OR* = 0.49) education had significantly lower odds of reporting good health. Hindu participants, compared to non-Hindu, also had lower odds of reporting good health (*OR* = 0.10; 95% *CI*: 0.03–0.41). Interestingly, compared to those living in nuclear families, those living in multigenerational families had 44% lower odds of reporting good health after controlling for other variables in the model. Having one morbidity resulted in 48% lower odds of good health. Moreover, not surprisingly, having multiple morbidities led to 60% lower odds of good health.

## Discussion

The goal of this study was to assess the association between two measures of family support (i.e., assistance with various aspects of daily life and with activities of daily living) and older family members’ self-rated health. The initial findings supported both hypotheses, whereby family support was associated with higher odds of better health; however, after adjusting the model for sociodemographic characteristics, family support with various aspects of daily life only approached significance (*p* = 0.087).

These findings supported the hypothesis that older adults who received assistance with activities of daily living from their families had better self-rated health. Although this is the first research to examine this association among Nepali older adults, previous studies conducted in neighboring countries with similar socio-cultural contexts are informative. Studies conducted in India and China found that older adults with family support reported better self-rated health [11, 21, 59] and higher odds of suffering from chronic illness among those who lived alone compared to those who lived with their families [60].

Several factors, consistent with the functional and normative components of the intergenerational solidarity model, explain the observed positive role of family support for the well-being of older family members in this study. Family provides strong social, psychological, and financial support to older adults, positively impacting their physical and mental health [23, 61]. Family members can create a conducive environment for health promotion in several ways, including providing companionship to health facility visits, assuring adherence to healthy diets and medication, and providing personalized care during illness [62]. Specifically, adult children typically accompany their older relatives to hospital visits in Nepal [62]. Further, family assistance reduces stress through emotional support and facilitates access to formal and informal care for older adults [63, 64]. These family dynamics operate in Nepal.

The second hypothesis that older adults who received support with various aspects of daily life had better self-rated health was supported in the unadjusted model. However, it only approached statistical significance after controlling for socio-demographic variables. This could be because of the highly disproportionate distribution of data in some variables, for example, marital status, family structure, and religion, across the categories. Particularly, ethnicity seemed to influence the statistical significance of the adjusted model because when it was removed from the final analysis, the hypothesis was significant even when including other covariates, suggesting a strong confounding role of ethnicity.

In contrast to the above findings, family structure was associated with lower self-rated health. Notably, it was not significant in the simple regression. However, this study lacks contextual factors such as who older adults live with (i.e., their son’s or daughter’s family), whether older adults follow rotational living, and whether older adults left their original homes and migrated to live with their adult children. Earlier research in Nepal and India also has shown mixed results. A study conducted in Nepal showed no association between family structure and self-rated health [65]. Although Agrawal [60] concluded that living with family in India resulted in better health, another study from the same country showed that older adults who co-resided with their adult children had more depressive symptoms than those who lived alone [66]. Therefore, future studies should consider these gaps while studying the impact of family structure on older adults’ health.

### Limitations and strengths

As with all research, this research has limitations, including the possibility of social desirability bias, lack of causality, and lack of generalizability of findings to all Nepali older adults, especially those residing in urban areas. Although bilingual translators translated the tools into Nepali language, some linguistic and cultural nuances might not have been entirely retained, possibly reducing the content validity of this study. Perhaps more problematic is that, despite the global use of self-rated health in research, some researchers have raised concerns about using a single item to measure subjective health status in middle and low-income nations [5, 67]. This is because an individual’s social circumstances and the lack of medical services, particularly for underprivileged groups, may negatively impact how they perceive their health [5, 67]. Despite this concern, several demographic and population health surveys in low- and middle-income nations, notably India and Nepal, have successfully employed self-rated health in their research [61, 68–70]. Similarly, measurement bias cannot be ruled out in the measurement of the two predictors of interest based on dichotomous responses.

Measuring family support with two single items is not the most effective approach. Additionally, there is some conceptual ambiguity and potential overlap in the specific types of support covered by each item, such as assistance with daily life and activities of daily living. Measuring support solely through activities of daily living may not encompass all dimensions of social support and, in particular, may not effectively assess emotional support. However, although not explicitly defined, when family support living under the same roof in Nepal is discussed, there are aspects of emotional support intertwined with every kind of support provided to older family members. Nevertheless, it is also worth noting that this research marks an essential initial exploration into the relationship between family support and self-rated health in Nepal.

This study was conducted during the COVID-19 pandemic, which could have impacted people’s willingness and ability to participate in surveys or interviews. Additionally, the pandemic may have influenced health outcomes, and lockdown measures, which led to more time spent at home, may have provided family members with increased opportunities to care for and support older parents. As a result, there may be biases associated with participant selection and responses during this specific time frame. Future research could use multiple items to comprehensively assess the impact of individual and multiple sources of support identified in the intergenerational solidarity model of support.

Despite these limitations, there are several strengths to this study. Using a tablet-based questionnaire enhanced data collection by significantly reducing turnaround time, minimizing error, and lowering costs. The survey employed trained local health professionals with years of fieldwork experience, which helped improve the speed and accuracy of data collection. Despite being conducted during the onset of the COVID-19 pandemic, a large study sample was obtained.

Although Bengtson and colleagues’ intergenerational solidarity model has informed family support in different cultural contexts, with few exceptions [71], these have been predominantly in western cultures. Thus, future application of the intergenerational solidarity in developing countries could help document the evolving nature of family support in collectivist contexts where shared housing and filial piety are being affected by changing demographics, rural to urban migration and migration out of the country.

### Implications for policy and future directions for research

In terms of policy, this study suggests that a family’s influence on an older person’s health is important. Perhaps in large part, because Nepal does not have strong social security and health care policies, the family remains a crucial support system for their older members’ care. The Nepal government provides a nominal fund of US$ 30 as an old-age allowance, but this does not address many old people’s financial hardships [72]. In countries with low economies like Nepal, it is important to acknowledge and value family support and the family environment as central components of the nation’s health care and long-term care plan and policies to improve the holistic health and well-being of older adults’ care.

In this context, family care needs can be encouraged and reinforced through the provision of tax offsets and other benefits to informal/family caregivers, such as food subsidies, medical discounts, priority housing, and other supplementary attractions [73]. Such incentive programs may further cultivate relationships between older adults and informal/family care providers so that informal/family care providers might not feel that supporting older people is burden. This also could help address the abuse toward older adults that is evident in many countries [73, 74].

Concurrently, for older adults who do not have a family care provider, a community-integrated care approach where care and support for older adults is shared among community stakeholders is one potential alternative [75]. Taken together, this highlights the need to evaluate family-based models of care that integrate family support under larger social and community-based support networks. Developing these family and informal intergenerational exchanges of support in the context of rapid globalization and youth migration can help address the pressing social and emotional issues that confront populations globally.

With respect to research, future efforts could explore these associations among families more broadly in Nepal and other countries where filial piety is dominant. Guided by the intergenerational family solidarity model [34, 35], future research should explicitly explore the full range of support (e.g., affectual solidarity, associational solidarity, consensual solidarity, functional solidarity [practical and financial assistance and support between family members], normative solidarity and structural solidarity) that are exchanged among family members.

This study only looked at unidirectional support provision from adult children to their older parents at one point in time. Adult children, on the other hand, do benefit from their parents’ contributions throughout the journey to adulthood. In later life, grandparents can fulfill the roles of historian, mentor, role model and nurturer, and provide wisdom and help develop altruism in their grandchildren [76]. Such compensative transference is also seen as reciprocity or an effort to balance the relationship [71]. Therefore, future research should consider studying the bi-directional intergenerational transfers that co-residence provides to older parents and their adult children. Further, an interesting question for future research is the extent to which some sources of support could be provided by non-family, informal community support, and their impact on older and younger family members’ health.

In addition to using more comprehensive and valid tools to measure family support, including additional constructs in future research will make results more conceptually and policy- and service-relevant. Examples include the personal economic resources (e.g., pensions, old-age allowance, retirement benefits) that are available to older adults; including the different dynamics of support received from migrant adult children (e.g., remittances) and those who reside locally; and the support exchanges that occur in ‘skip-generational households,’ where adult children are absent, and grandparents assume the responsibility of caring for their grandchildren within the same household.

## Conclusion

The findings of this study suggest that family support is crucial for the health and well-being of Nepali older adults. Therefore, local, provincial, and federal governments should develop action-based programs to meet the social, economic, and health care needs of its burgeoning older population by recognizing the importance of family-based care, supporting family-based care, and mitigating the obstacles in providing these services through informal and formal partnerships. Further, insights on family support from this study can guide efforts to understand how cultural norms and values can influence the living arrangements of, and care for, older adults in a global context and how the transition from traditional care system pose a significant challenge, particularly in low-income countries where a robust social security and alternative care options are lacking. This information can help both researchers and policymakers better understand challenges faced by older adults in different parts of the world and develop effective policies and interventions to improve the quality of life for older adults and the families who are integrally involved in these efforts.

### Supplementary Information

**Additional file 1: Supplementary Figure A.** GIS map of Nepal Highlighting the Two Study Districts. **Supplementary Table 1.** Demographic and Socioeconomic Characteristics of Nepal and the Study Districts.

## Data Availability

The datasets used and/or analyzed during the current study are available from the corresponding author on reasonable request.
