# Correction: The role of family support in the self-rated health of older adults in eastern Nepal: findings from a cross-sectional study

**DOI:** 10.1186/s12877-024-04741-8

**Published:** 2024-06-05

**Authors:** Aman Shrestha, Saruna Ghimire, Jennifer Kinney, Ranju Mehta, Sabuj Kanti Mistry, Shoko Saito, Binod Rayamajhee, Deepak Sharma, Suresh Mehta, Uday Narayan Yadav

**Affiliations:** 1 Department of Sociology & Gerontology and Scripps Gerontology Center, Miami University, Oxford, OH USA; 2 Little Buddha College of Health Sciences, Kathmandu, Bagmati, Nepal; 3 Centre for Primary Health Care and Equity, University of New South Wales, Sydney, Australia; 4 School of Optometry and Vision Science, Faculty of Medicine and Health, UNSW, Sydney, Australia; 5 School of Health Sciences, University of Tasmania, Hobart, TAS Australia; 6 Koshi Province Ministry of Health, Biratnagar, Koshi Nepal; 7 National Centre for Epidemiology and Population Health, The Australian National University, Canberra, ACT Australia

**BMC Geriatrics (2024) 24:20**

10.1186/s12877-023-04619-1

After publication of this article [1], the authors reported that in the ‘Ethics approval and consent to participate’-section, the reference number of the Nepal Health Research Council was incorrectly given as'#150/2020’ but should have been ‘#114/2021P’.

The original article [1] has been corrected.
