# Supplementary material for: The role of family support in the self-rated health of older adults in eastern Nepal: findings from a cross-sectional study
Source: BMC Geriatr. 2024 Jan 4;24:20. doi: 10.1186/s12877-023-04619-1 (PMC10768249; doi:10.1186/s12877-023-04619-1)
Supplement: Supplementary file 1 — Additional file 1: Supplementary Figure A. GIS map of Nepal Highlighting the Two Study Districts. Supplementary Table 1. Demographic and Socioeconomic Characteristics of Nepal and the Study Districts. [file 12877_2023_4619_MOESM1_ESM.docx]

**Additional file 1**

**Supplementary Figure A.**

*GIS map of Nepal Highlighting the Two Study Districts.*


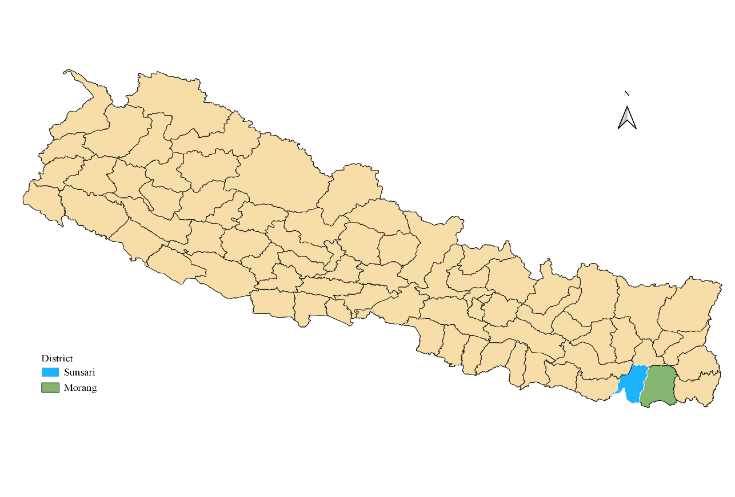


**Supplementary Table 1.**

*Demographic and Socioeconomic Characteristics of Nepal and the Study Districts.*

| Attributes | National | Study districts | |
| --- | --- | --- | --- |
|  |  | Morang | Sunsari |
| Total household number (in thousand) | 5427 | 214 | 162 |
| Total population (in thousand) | 26495 | 965 | 764 |
| 60+ population (in thousand) | 2154 | 80 | 57 |
| 60+ male (in thousand) | 1064 | 40 | 28 |
| 60+ female (in thousand) | 1090 | 40 | 29 |
| Aging index | 15.11 | 16.68 | 14.46 |
| Mean age at marriage (years) | 20.6 | 22.8 | 22.7 |
| Life expectancy at birth (years) | 66.6 | 67.3 | 67.2 |
| Literate population (6 years and above, %) | 66.6% | 70.9% | 68.9% |
| ^1^National rank of the district by literacy status | - | 25 | 33 |
| Urbanization level (%) | 29.0% | 44.2% | 34.3% |
| *Note*. ^1^National literacy rate urban: 82.3% and rural: 62.5%. In the eastern region, where the two study districts are located, rates are slightly higher (67.2%) than the national average. National literacy for the older population is low: 26.2% for ages 60-64 and 20.8% for 65+. Source: National Population and Housing Census 2011, Nepal | | | |
